# Targeting Cancer Stemness Using Nanotechnology in a Holistic Approach: A Narrative Review

**DOI:** 10.3390/pharmaceutics17030277

**Published:** 2025-02-20

**Authors:** Melinda-Ildiko Mitranovici, Laura Georgiana Caravia, Liviu Moraru, Lucian Pușcașiu

**Affiliations:** 1 Department of Anatomy, Faculty of Medicine, “George Emil Palade” University of Medicine, Pharmacy, Science and Technology, 540142 Targu Mures, Romania; liviu.moraru@umfst.ro; 2 Faculty of Medicine, “Carol Davila” University of Medicine and Pharmacy, 050474 Bucharest, Romania; laura.caravia@umfcd.ro

**Keywords:** nanotechnology, nanoparticles, stemness, stem cell-targeted treatment

## Abstract

Increasing evidence shows that a very small population of cancer stem cells (CSCs) is responsible for cancer recurrence, drug resistance, and metastasis. CSCs usually reside in hypoxic tumor regions and are characterized by high tumorigenicity. Their inaccessible nature allows them to avoid the effects of conventional treatments such as chemotherapy, radiotherapy, and surgery. In addition, conventional chemo- and radiotherapy is potentially toxic and could help CSCs to spread and survive. New therapeutic targets against CSCs are sought, including different signaling pathways and distinct cell surface markers. Recent advances in nanotechnology have provided hope for the development of new therapeutic avenues to eradicate CSCs. In this review, we present newly discovered nanoparticles that can be co-loaded with an apoptosis-inducing agent or differentiation-inducing agent, with high stability, cellular penetration, and drug release. We also summarize the molecular characteristics of CSCs and the signaling pathways responsible for their survival and maintenance. Controlled drug release targeting CSCs aims to reduce stemness-related drug resistance, suppress tumor growth, and prevent tumor relapse and metastases.

## 1. Introduction

In 1877, Virchow’s student Cohnheim discovered a new cell population in cancers called cancer stem cells (CSCs) which possess an embryonic profile [1,2] with therapy-resistant features and cause recurrence after treatment [1,3]. CSCs originate from adult tissue-resident stem cells, which are somatic stem cells that have been transformed under the influence of various genetic and epigenetic factors, or come from differentiated cells [1,4]. This specific subpopulation of cells accounts for about 2% of the tumor mass [4]. CSCs are characterized by a high degree of plasticity, changing their phenotype under chemotherapy and radiotherapy [1,3]. They possess the capacity for self-renewal with multidirectional differentiation and cancer progression due to their stemness property and interactions with the microenvironment, which allow for their survival, expansion, promotion of angiogenesis, and metastasis [1,5].

As a new therapeutic avenue in cancer management, the use of nanotechnology to eliminate CSCs has gained importance in the last decade.

Nanotechnology involves the synthesis of materials that manipulate matter at a very small scale—between 1 and 100 nm [6]. Considerable developments have been made in nanotechnology with the design of multiple nanoparticle platforms, including organic and inorganic nanomaterials, such as liposomes, dendrimers, polymeric systems, dendrimers, nano-emulsions, solid–lipid nanoparticles, metal oxide nanoparticles (gold, silver, iron, silica, titanium), and carbon nanotubes, along with RNA/DNA nanotechnology, quantum dots (QDs) and exosomes, small-molecule inhibitors, or surviving cell vaccination, combining chemotherapeutic drug delivery with photothermal strategies [6,7,8,9,10]. Nanocarriers possess the ability to encapsulate single or multiple drug molecules to enhance the delivery of poorly soluble drugs, overcoming the resistance mechanisms employed by CSCs. These abilities require control over the nanocarriers’ size and surface features [6,7,11]. Nanocarriers also possess a high surface area relative to their volume, which offers an enhanced half-life circulation time, improved tissue distribution, and decreased toxicity [6,12,13].

Traditional cancer therapies have failed, which leads to cancer recurrence and metastasis. These nanoparticles are capable of targeting metabolic pathways or cellular signaling pathways, CSC surface markers, and CSC survival-associated genes. They accurately target CSCs, accumulate the drug at the location, and maintain low cytotoxicity. In this context, nanomedicines is a promising tool in specific anti-CSC therapies [6,14,15].

The aim of our study is to highlight the importance of nanotechnology in the field of oncology, where the stemness features of certain tumor cells are responsible for tumor recurrence and metastasis, and the eradication of such cells is still elusory.

## 2. Data Search

Before starting the investigation, we decided on the most appropriate type of review for our study considering the holistic approach to the topic and based on the aim of our research and the main question, which is the usefulness of nanoparticles as a delivery platform for various drugs in cancer and whether they have applicability in stemness, which is the main cause of recurrences and metastases in cancer.

So, we are exploring the idea of finding an effective method for eradicating cancer with targeted treatment and minimal side effects. Initially, we discarded the hypothesis of conducting a scoping review, which starts from a broader question that generates hypotheses, whereas a systematic review focuses on a narrower niche and a rigorous analysis of data from the literature. However, we also leaned toward a scoping review due to the more descriptive elements in the literature generated by keywords, rather than a systematic review, where we would need to answer more analytical questions.

The hypothesis of an umbrella review was ruled out, as it is a high-quality systematic review of all systematic reviews and meta-analyses in the literature. First of all, nanotechnology is still a field with few medical applications and is continuously evolving, which requires close monitoring before an umbrella review can be conducted. Additionally, such a review requires a very rigorous methodology, appropriate software, bias reporting, transparent presentation of results, and, most importantly, a clear definition of the need for such an umbrella review.

Based on the heterogeneity of data in the literature and using keywords, we concluded that a narrative review is more appropriate, utilizing part of the methodology from a systematic review, including a wide range of studies and presenting the current status of the topics, highlighting novelties and future directions. A summary of the data with a meaningful synthesis could be conducted in this way.

This study was performed in 2024, using proper search mechanisms for specific keywords. Google Scholar and PubMed were chosen as the electronic databases for this search, from which we extracted 8130 titles. Editorials, reports, and case reports were excluded. On the basis of our inclusion criteria, full-text articles written in English describing clear and informative studies were sought, with the single exception of the Cohnheim study, which was not available in English [2]. Other articles for which only the abstract was available were removed. Duplicates were also excluded.

**Figure 1:**
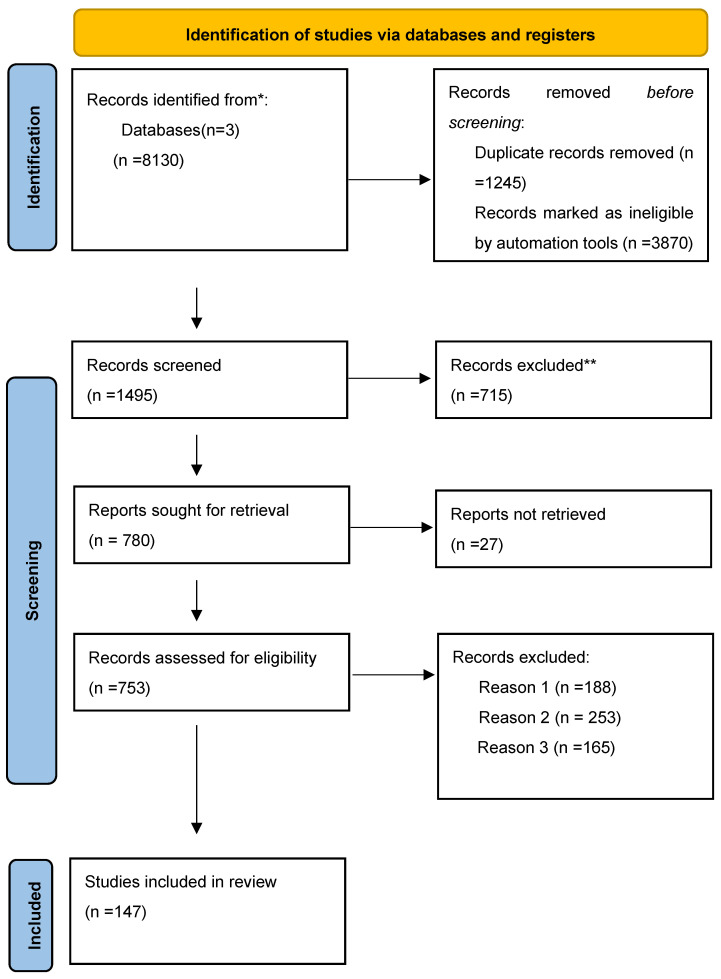
A flow diagram showing the selection mode [16]. * The number of records identified from the Google Scholar, Web of Science, and PubMed databases. ** Records excluded by a human. Reason 1: Records excluded as they were publications in a language other than English. Reason 2: Records excluded by a human reviewer due to inaccurate or inappropriate titles. Reason 3: Records excluded based on the study’s research design.

We took into consideration articles that provided new knowledge regarding targeting stemness using a nanotechnological approach; as such, 147 articles were chosen. A narrative approach was adopted due to the characteristics of data pooling. The selection mode is presented in a flow diagram (Figure 1).

We have structured our manuscript into sections about nanotechnology, the stemness property of cancer stem cells, and the targeting possibilities of stemness markers and pathways. We started with nanotechnology in order to find out if it has applicability in cancer and what its use entails. Then, we detailed stemness in cancer and how we could use nanoparticles, considering that CSCs are a small population of cells in a particular microenvironment.

## 3. Nanotechnology

Smart NPs are designed according to the locations and characteristics of tumors in order to ensure success against cancer. Among these NPs, carriers with a size of 10 to 100 nm and a rod-like or spherical structure are the most efficient [11,17,18]. The efficiency of NPs made of inorganic materials (such as gold) is better than that of NPs made of organic materials [11,19].

Nanotechnology has been developed to target CSCs, but it has some limitations. This is due to passive targeting based on the enhanced permeability and retention (EPR) effect [11,20,21]. Passive targeting refers to how drug-loading particles of certain sizes tend to concentrate in tumor tissues instead of normal tissues [22]. The EPR effect is typical of malignancies [22]. NPs can accumulate at tumor sites because of their lack of adequate lymphatic outflow. It is common practice to alter the size or form of NPs in order to facilitate greater passive accumulation at the tumor site [22]. The NPs’ antitumor efficacy after injection depends on the following stages: blood circulation, tumor accumulation, tumor penetration, cellular internalization, and drug release [11,23]. The delivery of targeted medication occurs through NP internalization due to receptor-mediated endocytosis. To facilitate this process, stimuli-regulated NP-releasing devices have been created. Such compounds react to inappropriate temperatures or internal stimuli such as redox mechanisms, biological particle overexpression, or pH modification. External stimuli affecting NPs can also be taken into consideration, such as magnetic fields, heat, ultrasound, microwaves, or light [22]. Further advancements in drug delivery systems using innovative stimuli-responsive biomaterials for target-specific release have emerged. Stimuli-responsive biomaterials are a novel platform providing greater bioavailability of anticancer molecules in the tumor microenvironment with lower systemic toxicity and wider biodistribution [24].

Several nanoparticles have been developed to target a number of cancer pathways and transcription factors involved in CSC stemness, such as NF-κB and HIF-alpha. These include silica nanoparticles, extravesicles, redox-sensitive nanoparticles, silver nanoparticles, gold nanoparticles, PLGA (poly (lactic-co-glycolic acid)) nanoparticles, polyethylene glycol–polylactic acid (PEG-PLA)-based nanomedicines, polyplex-based nanocarriers, shRNA nanoformulations, arsenic trioxide-based nanocarriers, solid lipid nanoparticles (SLNs), etc. [25].

Drug delivery can be enhanced by means of active targeting. In active targeting, NPs can be conjugated with aptamers, antibodies, peptides, and other small molecules [10,26].

In order to develop efficient targeted cancer therapies or diagnosis platforms, nanoparticles have also been combined with a variety of cells, such as immune cells, stem cells, bacteria, sperm cells, and adipocytes [27].

In the following, we discuss a few types of nanoparticles.

### 3.1. Organic Nanoparticles

Peptide-based vaccines capable of directly targeting the proteins expressed in tumor cells have emerged as a powerful but safe neoadjuvant immunotherapy. They have been most effective for patients in whom the body’s tolerance to cancer is minimized [28].

Poly(lactic-co-glycolic acid) nanoparticles (PLGA NPs) were investigated as a drug delivery system for paclitaxel (PTX), and the results showed that PLGA NPs enhanced the tumor suppression efficiency of PTX [29].

A poly(amidoamine) dendrimer can be used as an NP to deliver p70^S6K^ siRNA and protect the siRNA from degradation, thus targeting CSCs. For now, they have not been used in therapy as they lack specificity and have adverse side effects [30].

Sal-Docetaxel-loaded gelatinase-stimulating nanoparticles show promise as a new targeted therapy against CSCs with higher antitumor efficacy while also reducing side effects [31,32].

Cancer cell membrane (CCM)-coated nanoplatforms used to prepare a drug delivery system (DDS) for homologous tumor targeting demonstrated high efficacy due to their recognition and binding of tumor-specific receptors on the cell surface [33].

Lipid-based nanoparticles (LNPs) are also used to significantly enhance the effect and bioavailability of drugs while decreasing their side effects. LNPs can be liposomes, exosomes, nanocapsules, or nanospheres, all of which are used in biomedical applications [25].

Phytochemicals demonstrate considerable potential in nanotechnology. Phytochemical-based NPs can improve anticancer therapeutic effects and reduce the side effects of toxic chemotherapy [34].

Liposomes are nontoxic and biodegradable nanocarriers. They have the ability to stabilize compounds and widen their biological distribution while minimizing systemic toxicity [10,35,36]. To enhance their antitumorigenic effect, urokinase plasminogen activator (uPA) antibodies can be conjugated with liposomal nanobins to target the urokinase system, which is overexpressed in cancer cells but expressed at low levels in normal cells [26,37].

Polymeric micelles (PMs) represent a particular nanodrug delivery system that is widely used in oncology due to their high stability, increased cargo bioavailability, and biodegradability [38]. Cisplatin-loaded biodegradable copolymer nanoparticles (Cis-NPs) showed a protective effect on normal tissue; this was attributed to their ability to down-regulate the genes involved in chemoresistance, apoptosis, and cell proliferation, improving their efficacy [39]. Zileuton™ loaded on polymer micelles also reduced the amounts of circulating tumor cells and intra-tumoral cancer stem cells in breast cancer [40].

Using polyurethane–short-branch polyethylenimine, Lo et al. created a small-interfering-RNA nanoparticle that was able to block EMT and CSC-like cells’ radioresistance while also inhibiting Wnt signaling and targeting the Oct-4 genes up-regulated in certain cancers [41,42].

Hyaluronic acid (HA) and poly(lactic-co-glycolic acid) (PLGA) conjugated with polyethylenimine (PEI) were assembled to enhance stability. Hyaluronic acid (HA) was then used in nanoparticles to target CD44-positive cancer cells [43].

Recently, extracellular vesicles (EVs) have gained interest as a potential drug delivery system [44]. EVs are cell-derived nanostructures that have opened new avenues of targeted treatment in this field. They are particularly important in cell-to-cell communication [45]. For instance, exosomes loaded with anticancer drugs, such as anticancer peptides, nucleic acids, tumor-targeting proteins, and phytochemicals, are known to interfere with drug resistance pathways in several cancer cell lines [46]. CRISPR-Cas-based genome editing represents a powerful tool for modifying the genetic and epigenetic mechanisms necessary for treating many diseases. Until the discovery of EVs, researchers lacked a proper vehicle for delivering CRISP molecular machinery. EV carriers exhibit lower immunogenicity, reduced toxicity, and higher biocompatibility compared with classical drug delivery platforms such as lentivirus, adenovirus, or synthetic nanoparticles [44,47].

Another delivery platform was developed using antibodies for the selective delivery of chemotherapeutic agents. Antibody–drug conjugates have antitumoral effects per se and may also be modified to incorporate immunostimulatory agents, increasing their antitumorigenic activity [48].

Various immunotherapy approaches, such as dendritic cell (DC)-based vaccines, immune checkpoint inhibitors, adoptive T-cell therapy, and oncolytic viruses, are currently used to target CSC-associated cell surface markers [49].

Traditional stem cell-based gene therapies have failed because of their detrimental side effects. One novel application involves magnetic core–shell NPs for the delivery and activation of a heat-inducible gene vector using magnetic hyperthermia. The gene encodes TNF-related apoptosis-inducing ligand (TRAIL) in adipose-derived mesenchymal stem cells (AD-MSCs). These engineered AD-MSCs maintain their innate ability to differentiate and proliferate, making them ideal cellular carriers. The exposure of the engineered AD-MSCS to mild magnetic hyperthermia results in the selective expression of TRAIL [50]. Decidua-derived mesenchymal stem cells (DMSCs) have also been studied as a platform for carrying mesoporous silica NPs or doxorubicin-loaded NPs in cancer therapy to induce cancer cell death [51].

### 3.2. Inorganic Nanoparticles

Inorganic nanoparticles like silver nanoparticles (AgNPs), gold nanoparticles (AuNPs), mesoporous silica nanoparticles, and carbon-based nanoparticles are also employed as suitable nanoformulations in cancer diagnostics and treatment. AuNPs show specific characteristic features that allow them to access tumors and carry high drug doses [10,25]. However, researchers have encountered problems with them, such as weak stability, narrow biodistribution, and side effects. Moreover, CSCs can inhabit hypoxic areas, consequently avoiding agent delivery. Monoclonal antibodies can potentially be used for targeting CSCs [7]. Carbon-based NPs, specifically carbon nanotubes (CNTs), are employed as carriers because of their nano-size, capacity to enter cells, and multifunctional capacity [25].

Gold nanoparticles are a promising drug delivery platform that has gained researchers’ attention. An important side effect, however, is potentially toxic hyperthermia due to the heating of surrounding normal tissues [26].

Graphene oxide–silver nanocomposite may be a novel nanotherapeutic molecule for specifically targeting CSCs, revealing highly antitumorigenic features [52].

Superparamagnetic iron oxide nanoparticles (SPIONs) have been widely used in research on cancer therapy as a promising tool for targeted therapy. Their toxicity should be evaluated in detail [53,54].

Zinc oxide nanoparticles regulate the proliferation and invasion of cancer by means of miR-506-3p/CD164 signaling and are used to target cancer stem cells [55]. Mesoporous silica nanoparticles (MSNs) can improve drug effectiveness and reduce toxicity when loaded with 5-Fluorouracil (5-FU) for the treatment of gynecological cancers [56].

The application of smart nanotheranostics can eliminate CSCs, enhancing the survival of patients with malignancies and offering a better quality of life. Nanotheranostics is used to monitor the biodistribution of nanomaterials and view their intracellular localization in order to study complete patient response. It combines diagnostics with targeted treatment, sometimes associating organic and inorganic NPs. The development of smart nanotheranostics, with a superior ability to target CSCs [57,58], offers insights into the latest applications of these materials in cancer [59,60].

The nanoparticles that have been used to target cancer stem cells are presented in the table below (Table 1).

## 4. Stemness

In terms of the eradication of CSCs, traditional cancer therapies, including chemotherapy and radiotherapy, have major limitations, leading to cancer recurrence and metastasis. Targeting CSCs could therefore be a new therapeutic avenue [1].

Another survival mechanism of CSCs is their ability to evade attack by host immune cells, including CD8+ cytotoxic T cells and natural killer (NK) cells. CSCs are characterized by the expression of ABC multidrug resistance pumps (ABCB1, P-glycoprotein 1, and ABCG2). ABC transporters are a type of membrane efflux transporter with the ability to expel cytotoxic anticancer drugs from cells [4,11]. Efflux inhibitors associated with chemotherapeutic agents in nanoparticles (NPs) may allow for the co-localization of these molecules in tumor cells and limit their toxicities [34,61,62]. As a result, transport into the tumor is highly hindered. Several approaches, such as antiangiogenics [3] and modification of the tumor stroma [62,63], have been proposed to improve the intra-tumoral transport of drug carriers given the inadequate blood supply that is found in tumors associated with hypoxia [31]. Understanding the molecular mechanisms underlying cancers and CSC pathways highlights the importance of targeting CSCs in personalized medicine [1].

It has been shown that the chemoresistance of stem cells is basically linked with a frequent dormant state in low-proliferation-rate cells [26], hypoxic conditions [64], or, as shown above, the activation of drug efflux, such as ATP binding cassette (ABC) family transporters [26]. Another mechanism involved in chemoresistance is the overexpression of DNA repair mechanisms [65,66], such as base excision repair through increased poly (ADP-ribose) polymerase 1 (PARP1) activity [67] or decreased programmed cell death activity [68,69]. Last but not least, by targeting the acquisition of an epithelial-to-mesenchymal transition (EMT) phenotype and reversing this process, stem cells could be forced to differentiate and enter their dormant state [26]. In this context, many researchers have concentrated on finding stemness markers to target CSCs in order to kill or induce differentiation in the CSC population as a new therapeutic avenue against cancer and to avoid cancer resistance to classical therapy.

### 4.1. Targeting Surface Stemness Markers

Several surface markers, such as CD133, ALDH1/2, and CD44, have been used to identify CSCs [1]. The most frequently observed and studied surface marker of CSCs is CD133 [1,70], followed by CD44 [4,5,26]. CD133 is a glycoprotein characterized by five transmembrane domains that is present on the surface of tumor stem cells and has a critical role in maintaining stemness [10,35]. Specific antibodies against CD133 have been successfully developed [1,70]. Using antibody–drug conjugates [46] and monoclonal antibodies [71] inserted into drug delivery systems to target cancer cells crucially limits the systemic cytotoxicity to normal cells [35,72] during treatment [73,74] and leads to the development of a drug carrier [72], representing a promising approach for cancer treatment and diagnosis [61].

In preclinical studies, gold nanoparticles coupled with a peptide recognizing CD133 are one type of NPs used to target cancer stem cells [35,75,76]. Another type of NP is made with hyaluronic acid, which is a biocompatible linear polysaccharide with affinity to the CD44 receptor, thus being able to target cancer stem cells [26,77,78,79]. For example, Kesharwani et al. [80] designed a novel HA copolymer with the anticancer agent 3,4-difluoromethyl-curcumin and styrene maleic acid to form nanomicelles [10]. Lee et al. constructed a CD44-targeting nanoparticle containing ROS-cleavable thioketal-SN38-conjugated hyaluronan–cholesterol with photodynamic activation [81]. In photothermal therapy, light is transformed into heat. In a study by Wang et al. [82], a high-temperature breakdown approach was used [10].

According to other researchers, targeting CD44 is not particularly efficient [60]. This is why some researchers developed anti-CD44 antibody-modified superparamagnetic iron oxide NPs (SPIONPs). SPIONP-mediated hyperthermia can kill CSCs [83].

In addition, studies examining antibodies conjugated using liposomes as NPs showed that CD133-targeted Doxil had significantly higher cellular uptake, lowering the inhibitory doxorubicin concentration compared to that for non-targeted Doxil [35] as another novel surface stemness-targeted therapy.

Chen et al. developed a nanopore hydrogel to specifically target local CD133, and the hydrogel was shown to be effective in post-surgical areas [81].

Various other NPs have been studied to target CSC surface markers. NPs with SN-38 (anti-CD133 antibody-conjugated SN-38-loaded nanoparticles (CD133Ab-NPs-SN-38)), a topoisomerase inhibitor conjugated with anti-CD133 antibody, target CD133 + HCT116 cells [10]. Some researchers have used gold NPs loaded with cisplatin in ovarian cancer against the CD44 marker [60]. Wang et al. used CD133 monoclonal antibody loaded onto carbon nanotubes (CNTs) to destroy CSCs through photothermolysis [84].

Shah, Vatsal, et al., in their study, used polypropylenimine (PPI) dendrimer as a multidrug carrier loaded with paclitaxel as a cell death inducer, along with a synthetic analog of luteinizing hormone-releasing hormone (LHRH) peptide with tumor-targeting properties and siRNA targeted to CD44 mRNA. This nanotechnological system showed high therapeutic potential for the targeted treatment of ovarian carcinoma with a novel DDS that effectively and simultaneously transports a drug combination containing siRNA targeting CD44 mRNA and cytotoxic agents [85].

By targeting ALDH^+^CD133^+^ cells, graphene oxide–silver nanocomposite (rGO–Ag) enhances the cytotoxic and apoptotic potential of salinomycin in ovarian cancer stem cells and could become a novel nanotherapeutic molecule for specifically targeting and eliminating CSCs [60].

A novel polymeric nanoparticle encapsulating Hh pathway inhibitor (HPI)-1, a small-molecule inhibitor, was engineered by Chenna et al. [86]. HPI-1 was shown to have greater solubility and systemic bioavailability [86]. It was also effective against the population of CD133^+^-expressing cancer stem cells [87]. One of the difficulties encountered was specifically targeting CD133^+^ and destroying CSCs [88]. Thus, aptamers were used to target CD133. Ni et al. developed polymeric nanoparticles loaded with salinomycin and conjugated with CD133 aptamers (Ap-Sal-NP) to specifically target and destroy CD133^+^ cancer stem cells [88].

CD44 and CD133 are also expressed in many normal stem cells. Because of this, CD44- and CD133-targeted therapies might be challenging; further validation is required [81].

Some examples are presented in the table below (Table 2).

### 4.2. Targeting Oncogene Stemness Markers

Most cancer cells are characterized by genetic instability that leads to the development of malignant properties such as invasion, drug resistance, metastasis, and reduced apoptosis [89]. Frequent mutations occur in the genome, and cancer-related oncogenes are involved in this process. Blocking the activity of oncogenes can be useful as a specific targeted treatment for tumor cells [1]. Furthermore, oncogenes and their target cells interact, inducing proliferation and reprogramming of the epigenome through stem cell reprogramming. This could have a new role in the targeted treatment of CSCs against oncogenes [90] such as Oct-4 [4], SOX2 [11,31], and Nanog [11]. SOX2 is a multifunctional proto-oncogene that is closely associated with stemness and the epithelial-to-mesenchymal transition (EMT), while OCT4 plays a crucial role in the maintenance and restoration of CSC pluripotency [11].

These oncogenes facilitate the process of transforming somatic cells into a stem cell-like state, differentiating and self-renewing them into various cell types [11]. The characteristics of pluripotent cells are maintained by the pluripotency-associated oncogenes OCT4, Sox 9, and Nanog and also via the suppression of differentiation-related genes [5]. Moreover, the up-regulation of Nanog, Oct4, and Sox2 in CSCs inhibits apoptosis through various signaling pathways, such as the Sox2/ORAIL/STIM1 and Oct4/Tcl1/Akt1 pathways [11].

CSCs show increased multidrug resistance through the expression of ABC multidrug resistance pumps (ABCB1, P-glycoprotein 1, and ABCG2) [11]. In addition, CSCs undergo a persistent dormant state, which may contribute to therapy resistance. Multidrug resistance gene siRNA (*MDR1* siRNA)-based strategies have therefore been proposed [79].

Inorganic NPs, such as ferric oxide nanocubes loaded with small-molecule LDN193189, have been used against the stemness-related genes OCT4 (octamer-binding transcription factor 4) and Nanog [10].

Genotoxic agents trigger the DNA damage response, followed by cancer cell apoptosis. Because of their DNA repair potential, CSCs are resistant to DNA damage-induced cell death. The pathways used are ATM and CHK1/CHK2 phosphorylation or anti-apoptotic signaling pathways, such as WNT/β-catenin, Notch, and PI3K/Akt signaling. These pathways and oncogenes could be targeted by the PARP inhibitors (PARPis) used in gynecologic malignancies [91]. PARP inhibition with olaparib has been approved [92,93] in combination with cisplatin and is associated with very high response rates [94]. In a phase I study of radioresistant melanomas, the inhibition of multiple DNA repair pathways restored sensitivity to radiotherapy [95]. To date, specifically targeting DNA repair mechanisms has shown promise in preclinical studies on cancer stem cell-targeting treatments [26,93,96].

The most common strategies explored for overcoming chemoresistance include the use of nanoparticle carriers containing siRNAs to manipulate dysregulated genes [31,97]. New nanotechnological methods are applied using miR-155-5p mimics loaded onto chitosan nanoparticles to inhibit cell proliferation by regulating HIF1α expression [98].

Adipose mesenchymal stem cells with spatio-temporal magnetic core–shell nanoparticle (MCNP)-based delivery represent a novel drug delivery platform that activates TRAIL expression to enhance control over the activation of stem cell-based gene therapies [50].

Epigenetic programming is involved in genetic modifications and stemness enhancement. In this process, DNA methylation and histone acetylation play a key role; microRNA (miRNA) expression is also involved in cancer cells regaining stemness features [1]. DNA methyltransferase (DNMT) inhibitors are already being used in different types of malignancies [99,100] (Table 3). New combinations of commonly used drugs with epigenetic regulators (such as DNMT inhibitors, HDAC inhibitors, or miRNAs) to improve antitumorigenic efficacy have shown good results so far [31,100,101]. The microRNA (miRNA)-34a is used for tumor suppression as it modulates apoptosis and cancer stemness [42,56]. The replacement of onco-suppressor miRNAs provides an effective strategy against tumor heterogeneity [102]. But most clinical studies of antibodies such as bevacizumab conjugated with NPs and onco-suppressor miRNA were suspended due to their unfavorable results in early-phase studies [103,104]

### 4.3. Targeting Stemness Pathways

**Figure 2:**
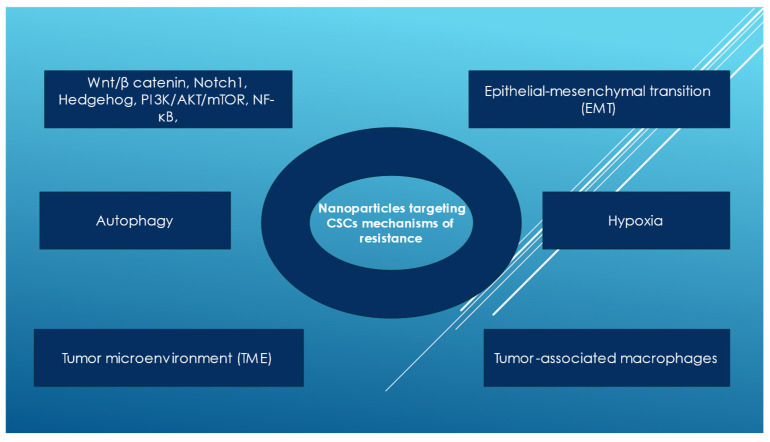
Nanoparticles targeting cancer stemness pathways.

The most common signaling pathways activated by CSCs are Wnt/β catenin, Notch1, and Hedgehog [1]. However, the STAT3, nuclear factor-κB (NF-κB), and phosphoinositide 3-kinase/AKT/mammalian target of rapamycin (PI3K/AKT/mTOR) pathways also regulate stemness properties in many cancers [4,5,26,105]. Iron plays critical roles in DNA synthesis and repair and in oxygen and electron transport [106]. However, free iron can catalyze the Fenton reaction, generating reactive oxygen species (ROS), followed by DNA damage. Intracellular iron accumulation has a metabolic impact on CSCs and the epithelial-to-mesenchymal transition [107,108,109]. The epithelial-to-mesenchymal transition has been targeted in preclinical models [26] to induce the differentiation of cancer stem cells in order to reduce stemness and restore chemo- and radiosensitivity [110,111,112]. Hence, inhibiting the EMT reduces CSC chemo- and radioresistance [10,31]. Gold nanoparticles (AuNPs), by reversing the epithelial-to-mesenchymal transition (EMT), inhibit ovarian tumor growth and metastasis in mice [60]. These nanoparticles can be loaded with the chemotherapeutic agent camptothecin and all-trans retinoic acid (ALTRA), which induces CSC differentiation though the EMT; studies in a mouse model showed the effectiveness of this drug combination [4]. Signaling stemness pathways are key mechanisms of cancer cell resistance, recurrence, and metastasis. They are exemplified below (Figure 2).

Notch signaling is a developmental pathway involved in embryogenesis. Its post-translational modifications lead to cancer development [41]. Notch inhibits apoptosis and enhances cell survival, self-renewal, and metastases [11]. Targeting the Notch3 pathway (a transmembrane protein) with Gamma-secretase inhibitors (GSIs) that are active against Notch receptors could serve as a novel therapeutic method in the effort to eradicate CSCs [1,113,114]. Inhibitors of the Notch pathway, along with other molecular agents such as miR-199b-5p and the γ-secretase inhibitor DAPT, are the basis of novel molecular targeted treatments [1,115,116].

Some researchers have designed CSC-targeted nanoparticles (NPs) against specific signaling pathways such as Notch, reactive oxygen species (ROS) signaling, or Wnt/β-catenin [1,117,118].

The Wnt signaling pathway is a developmental pathway involved in stem cell control. The Wnt family is a group of 19 glycoproteins in humans involving a complex mechanism of signaling pathways [41]. The Wnt signaling pathway is associated with the conversion of quiescent CSCs into active CSCs to promote cell cycle progression via β-catenin. Many proto-oncogenes activate the Wnt signaling pathway, modulating self-renewal and regulating CSC apoptosis and metastasis [11]. Through β-catenin signaling suppression by means of cytosolic phospholipase A2alpha (cPLA2α) ablation, we can obtain improved chemosensitivity in CC [119]. Attenuating β-catenin in vivo via integrin subunit alpha 5-targeting NPs is also a useful strategy for treating breast cancer [120].

In multiple-drug overloading of NPs, there is no guarantee that target tumor cells will receive optimal quantities of each drug simultaneously because their pharmacokinetic features differ, resulting in the outcome being compromised [121].

Another therapeutic avenue that has been investigated by researchers in order to target stemness is the tumor microenvironment (TME), which consists of tumor cells, tumor stromal cells, fibroblasts, endothelial cells, and extracellular matrix. The TME is infiltrated by immune cells such as macrophages and lymphocytes, which support the heterogeneity of the TME. The TME and its components promote drug resistance through a number of mechanisms such as cell-to-cell interaction and cell-to-EMT cross-talk. Hypoxia specific to the cancer environment creates an acidic state; additionally, the extracellular matrix and cytokines establish immunosuppression inside the TME [31,122].

The primary cancer and host cells create the TME and support the survival of CSCs by creating an imbalance between CSC differentiation and self-renewal [31]. They do this by stimulating signaling pathways such as Notch and Wnt, facilitating the evasion of CSC metastasis. CSCs can be indirectly targeted via components of the TME, such as tumor-associated macrophages (TAMs) or cancer-associated fibroblasts (CAFs) [123,124]. For example, miRNA125 delivered via TAM exosomes may suppress CSCs [31,125]. Other researchers used a nanoformulation of bola-amphiphilic dendrimer-encapsulated imatinib (Bola/IM) to target the CD117 marker of CSCs. This new formulation was reported to be effective in metastatic drug-resistant ovarian cancer cells, as compared to IM alone, through a novel and tumor-specific β-catenin/HRP2 axis [126].

The TME limits the delivery of chemotherapy to the cancer site, leading to therapy failure. Antiangiogenic therapy and immunostimulatory therapy have shown limited success due to a lack of drug penetration into the necrotic tumor core, associated with non-specific delivery or rapid elimination from serum, and dose-dependent toxicity. Nanoparticles that target the TME vasculature, the ECM, or distinct immune subpopulations through surface markers or stemness pathways are applied to overcome this issue [25,127].

Another important pathway in the stemness process is PI3K/ATK. Activation of the PI3K/ATK pathway promotes CSCs through the activation of HIF1α and HIF2α [128]. Hypoxia maintains CSCs in a dormant state with a low proliferation rate and is considered an independent predictor of disease progression, higher metastatic potential, and treatment failure [31,129,130].

Polymeric micelles (PMs) represent a particular nano-DDS that is widely used in the biomedical field due to their high stability, increased cargo bioavailability, biocompatibility, and biodegradability, helping to specifically eliminate the CSC subset. New therapeutic strategies have been developed based on the blocking of the molecular pathways essential for CSC survival, such as through the arachidonate 5-lypoxigenase (ALOX5) enzyme and the structural maintenance of the chromosome 2 (SMC2) protein. PMs were the nano-DDS used in these scenarios [38]. The pharmacological approach was implemented with the ALOX5 chemical inhibitor ZileutonTM, and the biotherapeutic approach was implemented with the antibody anti-SMC2 (Ab-SMC2) protein for specific SMC2 blockade. Pluronic^®^ F127-based PMs were designed to encapsulate ZileutonTM (PM-ZileutonTM) and Ab-SMC2 (PM-CON:SMC2), with increased biocompatibility, improved bioavailability, and low toxicity [38].

Another mechanism with a significant impact on the occurrence and development of tumors is autophagy. In eukaryotic cells, autophagy is an important mechanism that regulates growth, death, and catabolism, maintaining homeostasis during stress. Autophagy is often triggered by the inhibition of specific signaling pathways such as PI3K/AKT [11]. Nanoparticles (NPs) can induce autophagy in cancer cells [131].

Lipid droplets (LDs) are cytoplasmic organelles that are hypothesized to retain anticancer drugs, decreasing their efficacy. They appear in cancer cells due to reprogrammed lipid metabolism and have an important role in cancer resistance [132]. An important strategy in targeting CSCs is to understand lipid metabolism and how the regulation of autophagy influences CSC phenotype. This could be an attractive strategy to eliminate CSCs using therapeutic agents with an effect on the modulation of lipid metabolism [133].

To limit the cytotoxic impact of some new agents with combined cytotoxic and immunomodulatory effects, such as shikonin (SHK), polymeric nanoparticles (NPs) were developed to target the tumor microvasculature. Biodegradable NPs of poly(lactic-co-glycolic acid) (PLGA) loaded with SHK were developed, with their surface conjugated with the solubilizing agent polyethylene glycol (PEG) and tumor endothelial marker 1 (TEM1)/endosialin-targeting antibody [131].

The Hedgehog (Hh) pathway has a key role in controlling cell proliferation, embryonic development, and morphogenesis in animal development, but it is also expressed in adult tissues, being involved in tissue repair and regeneration. Defects in Hh signaling may affect tumor development and embryonic development [41]. Hedgehog enhances tumor growth and the development, maintenance, and regulation of residual CSCs after therapy [11]. Erythrocyte membrane-camouflaged PLGA nanoparticles were loaded with Cyclopamine, an inhibitor of the Hedgehog pathway, resulting in remarkable cell proliferation inhibition in vivo [134].

However, according to other studies, these agents could not reduce tumor progression, in addition to having serious adverse effects due to their pharmacokinetics profile [135].

NF-κB controls immune and inflammatory responses and enhances the expression of factors involved in angiogenesis or adhesion, such as IL-8, stemness oncogenes, and vascular endothelial growth factor [11]. Pompo et al. developed curcumin-loaded spherical PLGA NPs to suppress the NF-κB pathway in osteosarcoma stem cells [136].

Different inhibitors such as small molecules and antibodies have been used to inhibit the NF-κB pathway, but with unsatisfactory effects because of their low stability in blood circulation and poor cellular internalization [137].

Tumor-associated macrophages (TAMs) have been proposed as a therapeutic target to reduce resistance to antiangiogenic therapy in cases of tumor stemness. Ovarian TAMs can be selectively targeted using G5 dendrimer nanoparticles loaded with methotrexate as both a ligand and a toxin. G5 methotrexate (G5-MTX) nanoparticles deplete TAMs in cancer [17].

Despite initial expectations, the clinical effectiveness of some nanoparticles such as dendritic cells has not been as significant as anticipated [11]. Some of these nanoparticles are presented in the table below (Table 4).

## 5. Conclusions and Future Directions

The use of nanoparticles has the potential to have a major impact on cancers in terms of diagnosis and treatment. NPs are developed to deliver drugs using various mechanisms—passive targeting, active targeting, solubilization, and triggered release—which increase efficacy and decrease side effects. There are many challenges in the clinical translation of NPs, such as safety, biocompatibility and cost-effectiveness. There are limitations in declaring NPs efficient or not in clinical use [138].

However, nanotechnology has led to the development of promising solutions via the introduction of innovative tools and techniques that facilitate targeted drug delivery [139,140]. But, for the majority of nanocarriers, the therapeutic effects are compromised by either poor tumor accumulation or inefficient cellular internalization [141].

There is an urgent need to improve NPs’ performance because several issues have been encountered. A SWOT analysis is presented below, regarding NP development and their use in cancer [Table 5].

For example, protein coronae can develop on NPs’ surfaces after they are injected, which changes their interactions with the environment and attracts phagocytic cells that eliminate them. Heterogenous vascularity also affects their distribution within tumors, and their penetration is affected by the dense extracellular matrix. Cell membranes can also be an obstacle for NP internalization [11].

For this reason, we recommend the creation of interdisciplinary research collaborations among biological, clinical, and pharmaceutical scientists in association with scientists in engineering, physics, and chemistry. The focus should be on training (bio)medical researchers in the field of nanotechnology and creating nano-based solutions [142]. The study of nanomedicines should focus on increasing their bioavailability and reducing their damaging side effects. Additionally, the use of a smaller number of medications would result in cost reductions [143].

Recent advances in nanotechnology have enhanced the development of biodegradable nanocarrier delivery systems and exosomes with increased drug availability and wider distribution, allowing for multimodal therapeutic systems including photodynamic strategies and immune vaccination. Targeting tumor-selective inhibitors of apoptosis proteins has shown great success in preclinical studies, but more research is needed to show a benefit in clinical trials [8]. In addition, tumor cell-specific antibodies loaded onto NPs’ surfaces will pave the way for the development of more tumor-specific vehicles to target CSCs. Further modifications of nanocarriers are required before these strategies can enter clinical validation [8]. Additionally, most nanovehicles enter the cytoplasm rather than the nucleus, where anticancer drugs are most effective [8]. An example of a multitasking platform utilizing NPs is the combination therapy developed by combining gold nanorods as caps conjugated onto a mesoporous silica shell with synergistic photothermal and chemothermal therapy. NPs constructed with a modular strategy can be rapidly engineered with potential intracellular delivery of several therapeutic factors.

Sonication-assisted layer-by-layer (SLBL) technology was developed to obtain an efficient drug delivery platform by controlling the nanocolloid size to within 100–300 nm, increasing the drug content (70% wt), enhancing shell biocompatibility and biodegradability with controlled release, and producing multidrug-loaded systems. Stable nanocolloids of paclitaxel (PTX) and lapatinib were prepared via the SLBL method and have shown efficacy in chemoresistant ovarian cancer cells [144]. Various other categories of nanocarriers have been employed, including lipid, protein, polymeric, solid nanoemulsion, and hybrid systems [145]. Multitasking platforms utilizing NPs could become a viable solution. To assess biodistribution following the administration of NPs, histology is used, which is a cost-effective technique. Electron microscopy can provide more detailed information under very high magnification [146,147].

**Table 5:** SWOT analysis of NP use in cancer.

**Strengths**	**Opportunities**
- very small-sized particles encapsulate therapeutic compounds [11,139,140]. - overcome barriers in theory [11,138]. - target specific organs [11,144,145]. - reduce drug toxicity [139,140].	- target CSCs, with the possibility to overcome relapses and metastasis, enhancing curative outcomes [139,140]. - translational assessment of nanomedicine by determining the biodistribution of NPs [144]. First mechanism: enhance blood circulation of NPs by modifying their size [11,139]. Second mechanism: tumor accumulation [11,143]: - through small size of particles. - through stimuli-responsive NPs which respond to external stimuli that have an influence on tumor accumulation. - sequential drug delivery. Third mechanism: cellular internalization [11,141]: the development of an enzyme-sensitive cell-penetrating NP. Fourth mechanism: targeting and penetration [11,141]: - through antibodies which can be attached to a surface ligand. - by avoiding uptake by normal cells. - through aptamers which possess high stability and low immunogenicity. - regarding NPs that can remain trapped in membranes, through endosomal escape via improved gene and protein delivery systems. Fifth mechanism: drug delivery [8,11]: NPs have to target specific sites such as mitochondria or nuclei, which can be achieved by targeting pathways where drugs can be taken up into the cells. Multitasking NPs seem to be the ideal delivery platform.
**Weaknesses**	**Threats**
- impaired effectiveness due to various biological barriers; attracting plasmatic proteins on their surface which limits their interaction [8,11]. - eliminated by phagocytic cells [11]. - TME with heterogenic vascularity which restricts delivery [11]. - during internalization, NP penetration into cells could be obstructed by membranes [8,11,141].	- toxicity to normal cells [8,11]. - lack of targeting of specific cells [11]. - pharmacokinetic differences between the drugs overloaded on NPs in multidrug delivery systems [11,138]. - controlling the time between NP circulation and their cellular internalization can be challenging [11,139]. - CSC recognition and subsequent elimination is also a challenge [11].

A deeper understanding of the biological interactions, release of drugs, and dose response effects will allow for the development of nanotherapeutic platforms with higher efficiency and safety. The utilization of computer simulations and mathematical modeling is of great importance in the development of nanomedicines for personalized therapy. One of the major obstacles is their heterogeneous nature, which depends on the NPs’ characteristics. Identifying significant obstacles in this complex will allow for the development of novel approaches to overcome them [11].

Each individual has unique characteristics and exhibits different responses to treatments, an essential factor to be acknowledged. Personalized medicine becomes the optimal approach in the field of cancer treatment. The identification of CSC markers could be helpful in designing innovative NPs loaded with anti-CSC agents. However, a deeper understanding of CSC behavior and several issues related to NP development must be addressed in order to overcome existing challenges.

## Figures and Tables

**Table 1:** Nanotechnology used in oncology to target stem cells.

Article	NPs	Effect Type	Mechanism	Side Effects
[28]	Peptide-based vaccines	Neoadjuvant immunotherapy	CD8 T-cell responses	Injection site reactions, headache, fatigue
[29]	Poly(lactic-co-glycolic acid) nanoparticles (PLGA NPs)	Drug delivery system for paclitaxel (PTX)	Limits expression of chemo-resistant genes (*ABCG2* and *MDR1*)	Safe carrier
[30]	Generation 6 (G_6_) poly(amidoamine) dendrimer	Drug delivery system for p70^S6K^ siRNA	Protein kinase p70 S6 kinase (p70^S6K^), an effector of the phosphatidylinositol 3-kinase/Akt pathway, downstream effector of mTOR	Potentially adverse side effects
[32]	Sal-Docetaxel-loaded gelatinase-stimulus nanoparticles	Salinomicyn	Targets CSCs	Reduced side effects
[25]	Liposomes	Carrier of paclitaxel	Overexpresses ICAM-1 molecular target	Reduced side effects
[35,60]	Gold nanoparticles	Multidrug carriers		Hyperthermia
[53]	Graphene oxide–silver nanocomposite	Salinomycin	Specific targeting of highly tumorigenic ALDH^+^CD133^+^ cells and elimination of CSCs	
[54]	Iron oxide nanoparticles	miR-21 inhibitor loading	Mitogen-activated protein kinase signaling pathway	Toxicity should be evaluated
[55]	Zinc oxide nanoparticles		miR-506-3p/CD164 signaling	
[10]	Liposomes	Paclitaxel and salinomycin	Apoptosis, autophagy	Minimized systemic toxicity
[38,39,40]	Polymeric micelles (PMs)	Increased cargo bioavailability	Down-regulates genes involved in chemoresistance, apoptosis, and cell proliferation	Low-level side effects, biodegradability
[41]	Liquid–lipid nanoparticle delivery system	Cyclopamine	Reported slow tumor growth	Not available
[41,42]	Polyurethane–short-branch polyethylenimine		Aldehyde dehydrogenase 1+/CD44+ CSC-like cells; inhibits Wnt signaling	
[57,58,59]	Nanotheranostics	Drug delivery	Biodistribution of nanomaterials; targets CSCs	Not available
[39]	Low-molecular-weight glucosamine/L-lactide (GluN-LLA) and poly-L-lactide/PEG (PLA-b-PEG) biodegradable copolymers	Cisplatin	Protective effect of nanoparticle system according to its ability to down-regulate several genes involved in chemoresistance, cell proliferation, and apoptosis	Good compared to cisplatin
[43]	Poly(lactic-co-glycolic acid) (PLGA) conjugated with polyethylenimine (PEI)	Drug delivery with greater stability	CD44-positive cancer cells	Not available
[44,45,46]	Extracellular vesicles (EVs), cell-derived nanostructures	Anticancer peptides, nucleic acids, tumor-targeting proteins, phytochemicals	Interferes with drug resistance pathways to carry drugs modifying genetic and epigenetic mechanisms	Lower immunogenicity, reduced toxicity
[44]	Lentivirus, adenovirus	Anticancer drugs		Many side effects
[48]	Antibody–drug conjugate-based formulations	Chemotherapeutic agents	Still under clinical research; antibodies also have an antitumor effect per se	Under investigation
[50]	Engineered adipose-derived mesenchymal stem cells (AD-MSCs) and magnetic hyperthermia	Drug delivery	Oncogene targeting	Safer than conventional oncogene targeting
[51]	Decidua-derived mesenchymal stem cells	Carries mesoporous silica NPs or doxorubicin-loaded NPs	Cancer cell death	
[24]	Stimuli-responsive biomaterials	Drug delivery	Various	No systemic toxicity

**Table 2:** Examples of nanoparticles targeting surface stemness markers.

Nanoparticles	Stemness Surface Marker	Mechanism of Targeting Markers
hyaluronic acid [26,77,78,79,80,81]	CD44	Biocompatibility with CD44
anti-CD44 antibody-modified superparamagnetic iron oxide NPs SPIONPs [83]	CD44	Antibody
doxil (a liposome NP) [35]	CD133	Antibody
nanopore hydrogel [81]	CD133	Biocompatibility
polypropylenimine (PPI) dendrimer as a multidrug carrier [85]	CD44	siRNA targeting of CD44mRNA
polymeric nanoparticles loaded with salinomycin and conjucated with CD133 aptamers (Ap-Sal-NP) [88]	CD133	Aptamers (stimuli-responsive NPs)

**Table 3:** Example of nanoparticles targeting oncogenes.

Nanoparticles	Oncogene Stemness	Mechanism of Targeting
ferric oxide nanocubes [10]	OCT4	Small molecules (stimuli-responsive NPs)
chitosan nanoparticles [98]	Genes (OCT4, SOX)	siRNA
DNA methyltransferase (DNMT) inhibitors conjugated with NPs [99,100]	Genes (OCT4, SOX, or other genes)	Epigenetic manipulation

**Table 4:** Examples of nanoparticles targeting stemness pathways.

Nanoparticle	Stemness Pathways	Mechanism of Targeting
gold nanoparticles (AuNPs) [60]	EMT	Reversal of EMT
integrin subunit alpha 5-targeting NPs [120]	Wnt/beta-catenin	Beta-catenin attenuation
tumor-associated macrophage (TAM)-based exosomes [31,125]	Hypoxia/TME through Wnt and Notch	miRNA delivery
(Bola/IM)—bola-amphiphilic dendrimer (Bola)-encapsulated imatinib (IM) [126]	Beta-catenin	Beta-catenin attenuation
polymeric micelles (PMs) [38]	Blocking of molecular pathways essential for CSC survival through structural maintenance of chromosome 2 (SMC2) protein	arachidonate 5-lypoxigenase (ALOX5) enzyme
poly(lactic-co-glycolic acid) (PLGA) loaded with shikonin SHK [131]	Targeting of tumor microvasculature	(TEM1)/endosialin-targeting antibody
G5 dendrimer loaded with methotrexate (G5-MTX) nanoparticles [17]	TAM	Deletion of tumor macrophages
NPs [130]	Autophagy	Induced directly
erythrocyte membrane-camouflaged PLGA nanoparticles were loaded with Cyclopamine [134]	Hedgehog pathway	Biomimetic inhibition
curcumin-loaded spherical PLGA NPs [136]	NF-κB pathway	Impairing the pro-tumor activity of acid-stressed mesenchymal stem cells

## Data Availability

No new data were created.
